# Environmental factors influencing red knot (*Calidris canutus islandica*) departure times of relocation flights within the non‐breeding period

**DOI:** 10.1002/ece3.10954

**Published:** 2024-03-05

**Authors:** Evy Gobbens, Christine E. Beardsworth, Anne Dekinga, Job ten Horn, Sivan Toledo, Ran Nathan, Allert I. Bijleveld

**Affiliations:** ^1^ Department of Coastal Systems NIOZ Royal Netherlands Institute for Sea Research Den Burg Texel The Netherlands; ^2^ School of Biological and Environmental Sciences Liverpool John Moores University Liverpool UK; ^3^ Blavatnik School of Computer Science Tel‐Aviv University Tel Aviv Israel; ^4^ Movement Ecology Laboratory, The Alexander Silberman Institute of Life Sciences The Hebrew University of Jerusalem Jerusalem Israel

**Keywords:** ATLAS, biotelemetry, departure decisions, movement ecology, non‐migratory flight, shorebirds

## Abstract

Deciding when to depart on long‐distance, sometimes global, movements can be especially important for flying species. Adverse weather conditions can affect energetic flight costs and navigational ability. While departure timings and conditions have been well‐studied for migratory flights to and from the breeding range, few studies have focussed on flights within the non‐breeding season. Yet in some cases, overwintering ranges can be large enough that ecological barriers, and a lack of resting sites en route, may resist movement, especially in unfavorable environmental conditions. Understanding the conditions that will enable or prohibit flights within an overwintering range is particularly relevant in light of climate change, whereby increases in extreme weather events may reduce the connectivity of sites. We tracked 495 (*n* = 251 in 2019; *n* = 244 in 2020) overwintering red knots (*Calidris canutus islandica*) in the Dutch Wadden Sea and investigated how many departed towards the UK (on westward relocation flights), which requires flying over the North Sea. For those that departed, we used a resource selection model to determine the effect of environmental conditions on the timing of relocation flights. Specifically, we investigated the effects of wind, rain, atmospheric pressure, cloud cover, and migratory timing relative to sunset and tidal cycle, which have all been shown to be crucial to migratory departure conditions. Approximately 37% (2019) and 36% (2020) of tagged red knots departed on westward relocation flights, indicating differences between individuals' space use within the overwintering range. Red knots selected for departures between 1 and 2.5 h after sunset, approximately 4 h before high tide, with tailwinds and little cloud cover. However, rainfall and changes in atmospheric pressure appear unimportant. Our study reveals environmental conditions that are important for relocation flights across an ecological barrier, indicating potential consequences of climate change on connectivity.

## Graphical abstract

The timing of departure is crucial for flying species, with adverse weather impacting flight costs and navigation. Our study on 495 overwintering red knots in the Dutch Wadden Sea revealed that approximately 37% (2019) and 36% (2020) undertook relocation flights within their overwintering range, where timing was influenced by factors such as sunset timing, high tide proximity, tailwinds, and cloud cover. Understanding departure conditions is essential for comprehending the ecology of migratory species and the connectivity of their overwintering habitats.

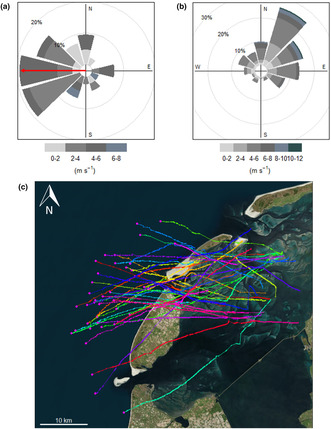

## 1 INTRODUCTION

The motivation of animals to move is often linked to changes in their internal state (e.g. hunger or reproductive state) and to external factors such as food availability or predation pressure (Morales et al., 2010; Nathan et al., 2008, 2022). Their movements can range from long‐distance seasonal migration across continents to local movements within their home range (Piersma et al., 2022; Somveille et al., 2015). Factors influencing the timing of animal movements vary across scales and might be driven by seasonally fluctuating resources or by daily changes in environmental factors (Mueller et al., 2011; Piersma et al., 1990). For example, caribou (*Rangifer tarandus*) and dusky warblers (*Phylloscopus fuscatus*) migrate across long distances every year, but departure times from the breeding grounds differ between years and largely depend on snowfall conditions for caribou (Le Corre et al., 2017), and precipitation and temperature for dusky warblers (Bozó et al., 2018). The timing of large‐scale movements is of particular importance for flying animals that cross large ecological barriers such as deserts, mountain chains, and large waterbodies, as they cannot stop or rest if adverse weather conditions arise (Anderson et al., 2019; Leyrer et al., 2009; Manola et al., 2020; Newton, 2007).

Studies on migrating birds revealed that weather, solar, and food conditions are important in determining departure timing, due to their effects on flight costs (Alerstam, 2009), the ability to navigate (Manola et al., 2020; Packmor et al., 2020), and the optimization of fuel stores (Hedenström & Alerstam, 1997). Adverse weather conditions such as strong headwinds or heavy rainfall can drastically increase the costs of flying (Hedenstrom & Alerstam, 1995) and even mortality (Anderson et al., 2019; Leyrer et al., 2009; Loonstra et al., 2019; Newton, 2007). Through changes in atmospheric pressure, certain bird and butterfly species can anticipate near‐future adverse weather conditions and delay departure appropriately (Breuner et al., 2013; Cooper et al., 2023; Manola et al., 2020; Packmor et al., 2020; Reppert & de Roode, 2018; Woodworth et al., 2015). Conversely, favorable tailwinds can reduce both time and energy used in flight (Butler et al., 1997; Kemp et al., 2012). The importance of the visibility of the setting sun (during evening departures) and stars (during the night) has been reflected in migratory departure timing decisions across species, resulting in a preference for low cloud cover (Manola et al., 2020; Packmor et al., 2020; Piersma & Jukema, 1990). Nocturnally departing birds prefer clear or partly overcast skies, as they use celestial cues from the stars and the setting sun for navigational purposes (Åkesson & Bianco, 2017; Packmor et al., 2020). Maximizing foraging opportunity before departure has also been found to be an influencing factor (Butler et al., 1997; Lank, 1989; Leyrer et al., 2009; Tulp et al., 1994). This is especially important for species that forage in intertidal zones where foraging patches are only exposed during low tide, making it favorable to leave during high tide when foraging opportunities are not present. However, the precise role of tidal cycles in departure decisions depends on the magnitude of tidal peaks and may vary per species and geographic location (Piersma et al., 1990; Piersma & Jukema, 1990; Tulp et al., 1994).

Many studies have demonstrated the importance of departure timing of long migratory flights (Alerstam, 2009; Anderson et al., 2019; Bradarić et al., 2020; Leyrer et al., 2009; Manola et al., 2020; Neima et al., 2022; Newton, 2007; Packmor et al., 2020; Piersma & Jukema, 1990; Sapir et al., 2011; Tulp et al., 1994; Woodworth et al., 2015), but non‐migratory flights, whereby birds may relocate within the overwintering range, have received little attention, even though distances traversed can extend dozens of kilometers (Frederiksen et al., 2012; Swindells, 2019) and can involve risky barriers. Given the potential rise in extreme weather events due to climate change, it becomes crucial to comprehend the specific conditions that birds prefer during departure, even for flights within their overwintering range. Many shorebird species' distributions in overwintering ranges, derived from bird count data, show that population numbers may be stable for the entire overwintering range but can vary in local areas, suggesting that large movements within wintering areas are common (Bijleveld et al., 2014; Evans, 2002; van Roomen et al., 2018). We will refer to these large‐scale movements of shorebirds within their non‐breeding or overwintering range as relocation flights. Relocation flights remain understudied, but given the dynamic distribution of many shorebird species throughout their overwintering period, it is likely that many species move large distances within their overwintering range (Evans, 2002). Local food conditions and individual differences in exploratory behavior may be causing these local fluctuations in bird numbers (Bijleveld et al., 2014; Rakhimberdiev et al., 2015). Whether birds select specific departure conditions for these relocation flights—that are relatively long but still shorter than migratory flights—within their non‐breeding range, remains unknown. By understanding how climatic conditions may enable or prohibit flights within an overwintering range of a species, we can study the connectivity of overwintering areas. In the face of climate change and increasing occurrence of extreme weather events, the role of specific weather conditions at departure may become even more important.

In this study, we investigated relocation flights of red knot (*Calidris canutus islandica*) from the Dutch Wadden sea towards the UK, which requires a 250 km flight across the North Sea. Red knot are migratory shorebirds, and the *islandica* subspecies is a Nearctic migrant that breeds in Eastern Canada and Greenland and overwinters along the coastline of Western Europe from July until May (Piersma et al., 1993). The Wadden Sea is a large overwintering area for red knots, but ringing studies indicate that some individuals ringed in the Wadden Sea also use foraging habitats outside of the Wadden Sea, such as the Wash in the UK within the same non‐breeding season (Bijleveld et al., 2014). Population numbers along the entire Western‐European coastlines remain relatively stable, while counts of overwintering red knots in local areas in the Wadden Sea show large fluctuations between years (van Roomen et al., 2018). Hence, a decrease in red knot numbers in the Dutch Wadden Sea, suggests an increase elsewhere along European coastlines and vice versa (Bijleveld et al., 2014; van Gils et al., 2006). These relocation flights are substantially shorter than an average migratory flight (>3000 km, from Greenland to the Wadden Sea), and therefore birds may be less selective of environmental conditions when deciding whether to depart.

Until recently, we lacked the technology to precisely track the timing of flight initiation of large numbers of individuals (Nathan et al., 2022). Here, we used a high‐resolution regional reverse‐GPS tracking system (ATLAS; Beardsworth et al., 2022; Bijleveld et al., 2022; Nathan et al., 2022) to identify departure times of red knots crossing the North Sea towards the British Isles. For those that departed on these relocation flights, we determined which conditions they selected for at their departure times and compared these to other available departure times. Specifically, we investigated the effects of wind, rain, atmospheric pressure, cloud cover, and timing relative to tidal cycle and to the solar cycle, which have all been shown to be crucial to migratory departure conditions (Åkesson & Hedenström, 2000; Alerstam, 2009; Bradarić et al., 2020; Butler et al., 1997; Lank, 1989; Leyrer et al., 2009; Manola et al., 2020; Packmor et al., 2020; Piersma et al., 1990; Sjöberg et al., 2017; Woodworth et al., 2015). We expected that birds would select for tailwind assistance, high atmospheric pressure, and no rain at flight initiation to decrease energetic flight costs; low cloud cover together with a departure time after sunset to increase visibility and the ability to navigate; and departure times close to high tide to maximize foraging opportunities on intertidal areas before departure.

## 2 METHODS

### 2.1 Tracking red knots

We caught red knots using mist‐nets on the Griend and Richel mudflats in the Wadden Sea, the Netherlands (53°15′ N, 5°15′ E), between July and October in 2019 (*n* = 251) and 2020 (*n* = 244). We equipped them with lightweight ATLAS tags (4.4 g, ~3.4% of their body mass), which we tracked with a high‐resolution regional‐scale reverse‐GPS tracking system (Beardsworth et al., 2022; Bijleveld et al., 2022; Weiser et al., 2016). The Wadden Sea ATLAS (WATLAS) tracking area is approximately 1400 km^2^ comprising a total of 26 receiver stations (Data S1) (Beardsworth et al., 2022, Bijleveld et al., 2022). Tags emit a radio signal at 434 MHz and if at least three receiver stations detect the transmission, the system can calculate a location based on the transmission time of arrival. If one or two receivers detect the tag, it will not be localized but a bird can still be detected in the tracking area (without a specific location estimate). Detecting birds on the outskirts of the tracking area is therefore still possible.

Tracking data were filtered and smoothed to improve data quality (Gupte et al., 2022). First, we removed all positions with speed >100 ms^−1^, as these are not realistic speeds for red knots (Battley et al., 2016). Second, position standard deviations of more than 150 m were also removed, as high values decrease the probability of a correct location. Finally, a median smoother of a moving window of 5 localizations was applied to smooth the tracks and to filter out possible errors that did not exceed our standard deviation and speed thresholds. These values were proven to be effective for filtering our movement data (Data S2) (Beardsworth et al., 2022).

### 2.2 Large‐scale non‐breeding relocation flights

**FIGURE 1:**
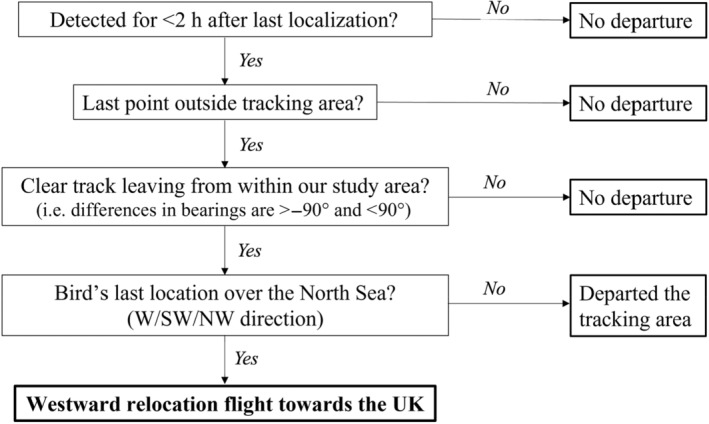
Decision tree used to determine whether birds had departed on a relocation flight based on four criteria.

**FIGURE 2:**
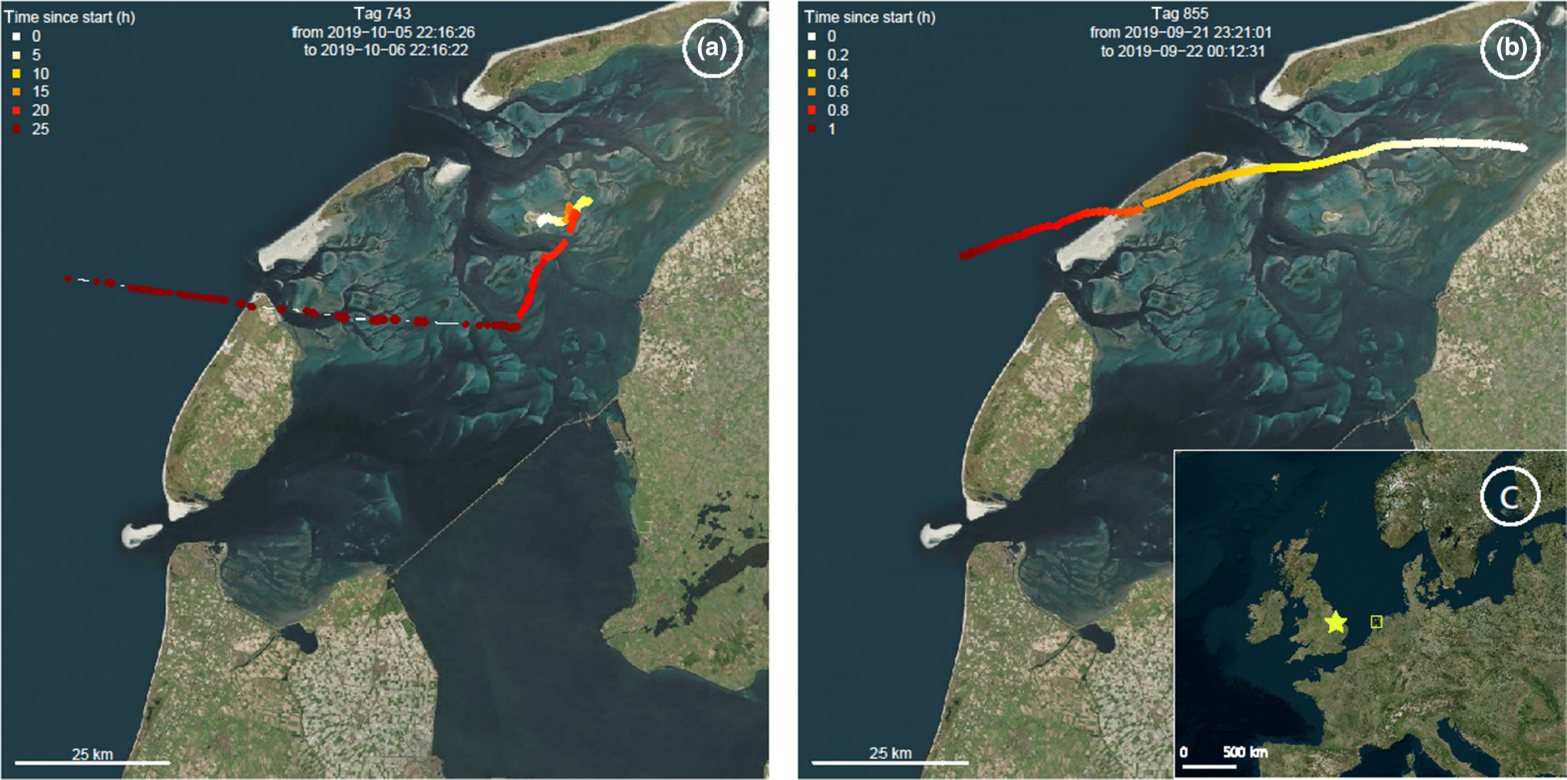
Movement tracks of red knots (a, b) in our study area in the Dutch Wadden Sea (yellow rectangle in (c)) departing on westward relocation flights towards the Wash, UK (yellow star in (c)). The colors of the tag represent the time differences between the movements: white indicates the start of the track, and dark red indicates the end of the track. Bird 743 (a) departs from within our study area, around the island of Griend. The departure location of bird 855 (b) remains unknown, as it enters our tracking area in a westward direction; hence, we are unable to identify the exact take‐off location and was therefore not included in our analyses. Background map © Microsoft Bing.

Relocation flights used in this analysis were selected using several criteria based on when the bird was last localized and detected (Figures 1 and 2). First, individuals cannot be detected for more than 2 h after their last localization. Detections indicate that a bird is still in our tracking area; hence if a bird truly left our tracking area, detections should end shortly after the last localization. Second, the bird's last location was outside the core tracking area (Figure 2a). Third, the movement track had to be a straight path leaving our study area, indicating goal‐directed transitory movement. We determined the straightness of the flight track by calculating the bearing between each localization in the last 20 min of a track. To ensure straightness, we used a threshold where the angles between consecutive localizations were below 90°. Tracks that departed from elsewhere (outside of our study area) but entered our tracking area en route were not included on our analyses (Figure 2b). Fourth, an individual's last known location was over the North Sea and it was moving in a westward direction (towards the UK).

It is challenging to obtain accurate numbers of birds that stay in the Dutch Wadden Sea throughout the non‐breeding season and birds that leave towards the UK because as the season progresses birds may also die and tags can fail or fall off, reducing our sample size and providing ambiguous data on the fate of the tagged individuals. To deal with this challenge, we assigned a cut‐off date representing the date at which 95% of all westward departing birds in our entire tracking dataset had departed and considered only tags that were still active at the cut‐off date. It should be noted that the proportions presented are not calculated by the total number of birds detected throughout the tracking season, but by those that were still detected after the cut‐off date.

### 2.3 Environmental variables

Cloud cover data were obtained from a Royal Netherlands Meteorological Institute (KNMI) weather station on Vlieland (53°15′ N, 4°55′ E, approximately 20 km west of Griend), at 10‐min intervals. Cloud cover data ranged from 0 (no cloud cover) to 8 (full cloud cover). Tidal data were collected by Rijkswaterstaat, from their station: West‐Terschelling (53°21.45′ N, 5°13.13′ E, approximately 12 km north of Griend) at 10‐min intervals, to which a 30 min‐lag was added, to obtain comparable tidal data for Griend. From these water levels, times of high tide and low tide were calculated. Departure times were matched to the closest high tide and differences in minutes before (−) or after (+) high tide were calculated. We also calculated how many minutes before (−) or after (+) sunset birds departed using the *sunrise*‐function from *maptools* (v.1.0‐2) package in R (v 4.2.1). Atmospheric pressure (mb), which was used to calculate changes in atmospheric pressure 1 h before departure times, and rain (0.1 mm/h), wind speed (ms^−1^) and direction (°) were obtained from a weather station on Griend (WeatherLink) at 1‐h intervals, approximately 1 meter above ground level. Using wind speed and direction, we calculated tailwind assistance, assuming full wind drift (Kemp et al., 2012) (Data S3).

### 2.4 Statistical analysis

To determine which environmental conditions are selected for when departuring on relocation flights, we used a resource selection function (RSF) to ask whether environmental conditions differed between actual departure times (used times) and randomly selected times (available times) (Matthiopoulos et al., 2020). We only analyzed departure from the Griend area to ensure that we could match departure times to weather conditions from that area. For every “used” departure time (*n* = 40), we randomly selected 30 “available” times (*n* = 1200), from a total of 96 h spanning the preceding 4 days to an individual's departure. The selection of 30 points was based on a sensitivity analysis (Data S4). We fitted a binomial generalized additive model (GAM), to account for non‐linearity, with a logit link function using the R‐package *mgvc* (v.1.8‐35). We assigned “used” times a weight of 1 and “available” times a weight of 1000 to avoid biased estimates of the resource selection function (Fithian & Hastie, 2013). After checking for co‐linearity, we included change (Δ) in atmospheric pressure, tailwind assistance, cloud cover, and year as linear fixed effects, and time relative to high tide and time relative to sunset as non‐linear fixed effects in our model. The resource selection functions, which are the exponent of the predictors from our model without the intercept, were scaled between 0 and 1 for visualization purposes.

## 3 RESULTS

### 3.1 Proportion of individuals that departed on relocation flights

All detected westward departures occurred between August 24 and November 14 in 2019 (*n* = 67), and October 2 and December 12 in 2020 (*n* = 40). The 95th percentile of departure dates differed between years: in 2019, 34 birds (37.0%) had departed on westward relocation flights on the cut‐off date of November 6, while 58 birds (63.0%) were still localized within our study area. In 2020, 27 birds (36.5%) had departed on westward relocation flights on the cut‐off date of November 29, compared to 47 birds (63.5%) that remained in our study area. In 2019, four more departures occurred after the cut‐off date, and in 2020 two more, resulting in a total of 67 red knots that departed on westward relocation flights across the North Sea in 2019 and 2020, 40 of which departed from the Griend area and were included in our resource selection function.

### 3.2 Conditions selected for at departure

**FIGURE 3:**
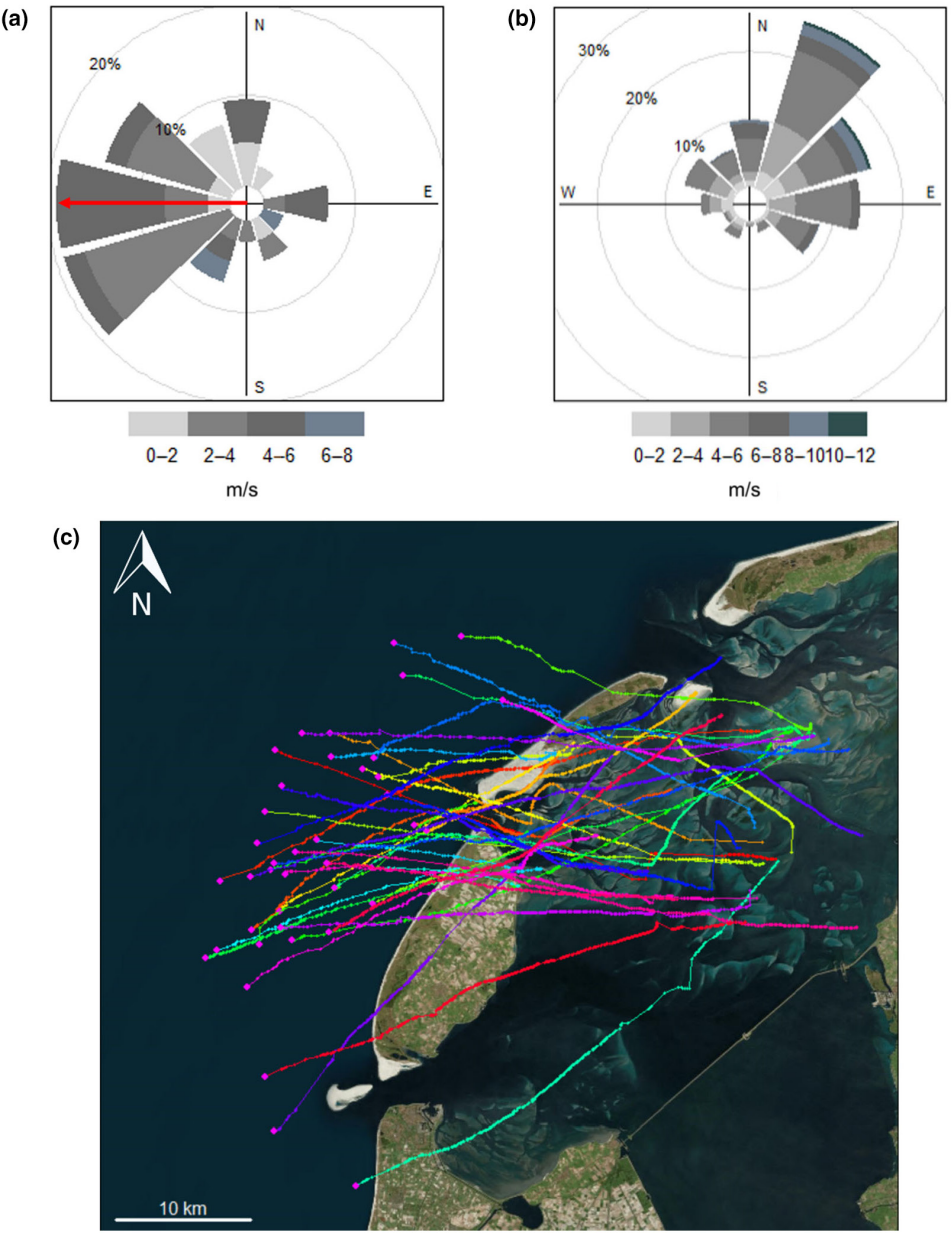
Wind speeds and directions selected for (a) at relocation flight departure times, where median flight direction is indicated with a red arrow. (b) Randomly selected non‐departure times within 4 days prior to the actual departure (available time‐points used in our analyses). (c) Departure tracks of the last hours of all westward flights used in this analysis. Background map © Microsoft Bing.

**FIGURE 4:**
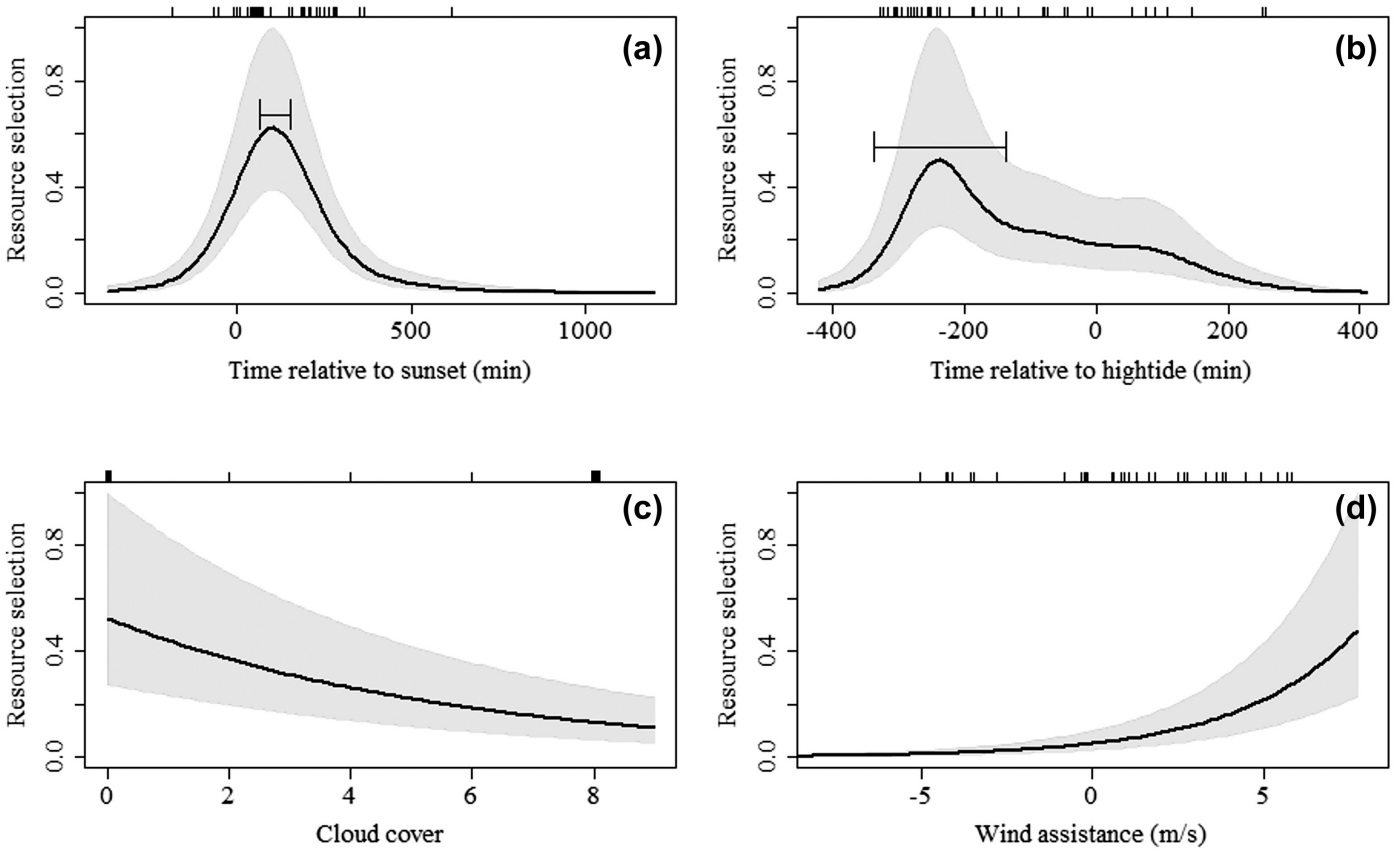
Resource selection functions of red knot departure times. Each panel shows the partial effect of one variable on the resource selection (*y*‐axis), which represents the chances of departure. The black line denotes the predicted partial effects of that variable on the chances of departure and the gray polygon represents one standard error. The black tick marks on the top *x*‐axis represent real departure times. Significant effects were found for (a) time relative to sunset (min), (b) time relative to hightide (min), (c) cloud cover, and (d) wind assistance (m/s). For the non‐linear variables, 95% CI of mean peak values are shown in the figure, when the model was bootstrapped 100 times. These resource selections are plotted as the scaled exponent of fitted logistic regression models after deducting the intercept. Non‐significant effects are not shown here.

Red knots selected flight departure times soon after sunset (*p* < .001, *μ* = 108 min, 95% CI [63, 152 min]) (Table 1; Figure 4a) and approximately 4 h before high tide (*p* < .001, *μ* = −238 min, 95% CI [−138, −337 min]) (Table 1; Figure 4b). They also selected for low cloud cover (*p* < .001, *β* ± SE = −0.197 ± 0.05) (Figure 4c), and tailwind assistance (*p* < .001, *β* ± SE = 0.28 ± 0.05) (Table 1; Figures 3 and 4d).

**TABLE 1:** List of covariates that were included in our binomial generalized additive model (GAM) with corresponding estimates (*β*), standard errors (SE), and *p*‐values (*p < .05).

	Covariates	*β*	SE	*p*
Linear components	Intercept	−12.89	1.01	.00*
	Year	−0.07	0.33	.83
	Wind assistance	0.28	0.05	.00*
	Cloud cover	−0.17	0.05	.00*
	Δ Atmospheric pressure	−0.27	0.34	.43
	Rain	−0.33	0.40	.40

	Covariates	Edf	Chi.sq	*p*
Non‐linear components	s (Time relative to hightide)	2.42	14.01	.00*
	s (Time relative to sunset)	3.30	26.39	.00*

## 4 DISCUSSION

Our study shows that red knots relocating from the Dutch Wadden sea to the UK select for departure times after sunset with tailwind assistance and low cloud cover. These requirements are similar to departures for longer migratory flights (Åkesson & Hedenström, 2000; Alerstam, 2009; Bradarić et al., 2020; Butler et al., 1997; Lank, 1989; Leyrer et al., 2009; Manola et al., 2020; Packmor et al., 2020; Piersma et al., 1990; Sjöberg et al., 2017; Woodworth et al., 2015), despite these relocation flights being much shorter (~250 km) than an average migratory flight of red knots (>3000 km). Birds chose favorable wind conditions (Figures 3 and 4d), which can minimize both time and energy expenditure on a flight (Alerstam & Lindström, 1990). They also selected for low cloud cover, most likely because celestial cues and landmarks will be visible during flight but, although we did not find precipitation itself to be important for departure, cloud cover is often correlated with the probability of precipitation, which may also be a reason for birds to select low cloud cover (Anderson et al., 2019; Leyrer et al., 2009; Newton, 2007). Additionally, there was a strong preference for departure times after sunset (Figure 4a), where 90.5% departed after sunset and 40.7% within 180 min after sunset. Departing after sunset can be beneficial as birds can use the polarized light at sunset for navigation, and celestial cues, such as the position of the setting sun and stars, when cloud cover is low (Åkesson & Hedenström, 2007; Lank, 1989). Nocturnal flights may also be associated with lower predation danger (Lindström et al., 2021), less turbulent wind conditions (Kerlinger & Moore, 1989), and reduction of water loss due to cooler and more humid air (Alerstam, 2009). Finally, we found that red knots choose departure times between 2.5 and 5 h before high tide, with a peak around −4 h (Figure 4b). The role of tidal cycles in migratory departures is generally explained by a maximizing foraging strategy, where birds make optimal use of the foraging time when food patches are exposed during low tide (Alerstam, 2009; Hedenström & Alerstam, 1997). Piersma et al. (1990).Personal field observations show that the magnitude of tidal peaks around Griend causes the foraging areas to flood approximately 3 h before high tide, which matches the preferred departure time in our results and thus supports a foraging maximization strategy.

Out of our 40 westward departing birds, 36 birds selected departure times with no rain and only 4 departed with rain (3 with 0.5 mm/h and 1 with 3 mm/h). Approximately 70% of the randomly selected (available) departure times also coincided with no rain and ~20% with very low precipitation rates of 0.5 mm/h. Overall, precipitation rates of >1 mm/h only occurred on ~10% of all available departure times. These low precipitation rates in both years may have affected our ability to measure the importance of rain in departure decisions. Like rain, changes in atmospheric pressure were not identified as an important variable for selecting a departure time. While atmospheric pressure has been described as a good predictor of migratory departures, this has mostly been described for songbirds, as atmospheric pressure can alter the activity of insects and therefore the foraging activity of insectivorous songbirds (Brust et al., 2023; Cooper et al., 2023; Packmor et al., 2020). In our study system with shorebirds who forage on benthic invertebrates in the overwintering grounds, atmospheric pressure might not be as important as it is for other species.

While it may seem logical for birds to always seek favorable conditions at departure times, and these seem to be similar for long and shorter flights, the relative importance of selecting favorable conditions could differ. Whether risks on days where not all environmental conditions are optimal, are weighed differently in relatively long (migratory) flights compared to shorter flights can give us insights into which environmental variables are most important in departure decisions and whether this changes with flight distance. From our formal analyses, it remains uncertain which environmental variable is most important for red knots when deciding when to depart, but investigating outliers, where conditions deviated from the majority of departure times gives us some insights. For instance, we observed three birds that departed before sunset, of which two were presented with unfavorable wind conditions (headwinds of up to 8 m/s) before their departure times. They departed at the first opportunity of tailwind assistance, suggesting they prioritize tailwind assistance over nocturnal departures (Data S5). Similarly, another study found that the *canutus* subspecies depart for migration from the Wadden Sea around sunset, but would delay their departures until morning to avoid adverse storm‐like weather conditions (Leyrer et al., 2009). Perhaps risking a navigational error on these relatively short flights, by not departing around sunset, outweighed the risk of spending too much energy on flying with headwinds.

In this study, we looked at environmental and climatic conditions as reasons to choose a departure time, but social information may also play a role in selecting a departure time (Piersma et al., 2020). Birds are more likely to set off in a flock, as flocking behavior can increase the chances of survival, especially if predation danger is high, decrease energetic costs and allows social information to be shared, thus making traveling together safer and preferred by many species (Beauchamp, 2004; Cresswell, 1994; Piersma et al., 1990). To set off together, a consensus must be reached about departing in that moment and this will likely be easier to reach in the most favorable conditions, regardless of travel distance.

### 4.1 Why relocate?

A decrease in local food availability and fast resource depletion rates can cause a proportion of red knots to temporarily emigrate out of the Dutch Wadden Sea to other non‐breeding grounds in the UK (Rakhimberdiev et al., 2015). We found that percentages of westward departing birds out of the Dutch Wadden Sea were similar between 2019 and 2020 (37.0% and 36.1% respectively). The date at which 95% of all westward departing birds had departed, however, was 23 days earlier in 2019 (November 6) than in 2020 (November 29). Variation in food stock depletion rates between years may cause this shift in departure dates. Relating food availability to relocation flights of red knots could help us better understand why these movements are happening. Additionally, Bijleveld et al. (2014) found that red knots that were ringed in the Dutch Wadden Sea had a higher probability of being resighted outside of the Wadden Sea if their gizzard mass, which is negatively correlated with exploratory behavior and diet (Ersoy et al., 2022), was relatively small. Thus, exploring whether individual differences in personality may explain why some individuals relocate and others do not, is worthwhile investigating. Understanding space use of a population and drivers of selecting or changing overwintering areas can be of importance for predicting the resilience of a population, especially in the face of climate change.

## 5 CONCLUSION

Relocation flights during the non‐breeding period are a largely understudied behavior, despite their potential significance for gaining a comprehensive understanding the ecology of migratory birds. Our findings show that the connectivity of mudflats is important for red knots during the non‐breeding period, and that climatic conditions at flight initiation matter. This is particularly relevant in an era of global climate change, sea level rise and anthropogenic changes to habitat. Our study contributes to building knowledge on how climatic conditions interact with ecological barriers to facilitate or restrict movement within the overwintering range.

## AUTHOR CONTRIBUTIONS

**Evy Gobbens:** Conceptualization (equal); formal analysis (lead); methodology (equal); writing – original draft (lead); writing – review and editing (equal). **Christine E. Beardsworth:** Conceptualization (equal); formal analysis (supporting); methodology (equal); supervision (equal); writing – review and editing (equal). **Anne Dekinga:** Data curation (equal); writing – review and editing (equal). **Job ten Horn:** Data curation (equal); writing – review and editing (equal). **Sivan Toledo:** Data curation (equal); resources (equal); software (lead); writing – review and editing (equal). **Ran Nathan:** Data curation (equal); resources (equal); software (supporting); writing – review and editing (equal). **Allert I. Bijleveld:** Conceptualization (equal); data curation (equal); formal analysis (supporting); funding acquisition (lead); methodology (equal); project administration (lead); resources (equal); supervision (equal); writing – review and editing (equal).

## FUNDING INFORMATION

This work was funded by NIOZ and the Dutch Research Council grand VI.Veni.192.051 awarded to AIB.

## CONFLICT OF INTEREST STATEMENT

The authors declare no conflicts of interest.

## Supporting information


Data S1–S5

## Data Availability

All data and materials have been made publicly available and can be accessed via Github: https://github.com/EvyGobb/Knot_departure‐conditions/tree/v1.0 and Zenodo: https://doi.org/10.5281/zenodo.7458323.
