# Supplementary material for: Environmental factors influencing red knot (Calidris canutus islandica) departure times of relocation flights within the non‐breeding period
Source: Ecol Evol. 2024 Mar 5;14(3):e10954. doi: 10.1002/ece3.10954 (PMC10915501; doi:10.1002/ece3.10954)
Supplement: Supplementary file 1 — Data S1–S5 [file ECE3-14-e10954-s001.docx]

**Supplementary Online Material for**

Environmental factors influencing red knot (*Calidris canutus islandica)* departure times of relocation flights within the non-breeding period.

Evy Gobbens*^1^, Christine E. Beardsworth^1,2^, Anne Dekinga^1^, Job ten Horn^1^, Sivan Toledo^3^, Ran Nathan^4^, and Allert I. Bijleveld^1^

^1^ Department of Coastal Systems, NIOZ Royal Netherlands Institute for Sea Research, NL-1790 AB, Den Burg, Texel, the Netherlands

^2^ School of Biological and Environmental Sciences, Liverpool John Moores University, Liverpool, L3 3AF, UK

^3^ Blavatnik School of Computer Science, Tel-Aviv University, Tel Aviv 67798, Israel

^4^ Movement Ecology Laboratory, The Alexander Silberman Institute of Life Sciences, The Hebrew University of Jerusalem, Jerusalem 91904, Israel

* Corresponding author e-mail: [e.gobbens@gmail.com](mailto:e.gobbens@gmail.com)

**Data S1**

*WATLAS Tracking System*

The Wadden Sea ATLAS (WATLAS) tracking area is approximately 1400 km^2^ comprising a total of 26 receiver stations (Figure 1) (Bijleveld *et al.* 2022). The tags emit a signal once every six seconds and if the tag is picked up by three or more receiver stations, the system can provide detailed movement tracks of red knots within our tracking area. If one or two receivers detect the tag, it will not be localized but a bird can still be detected in the tracking area (without a specific location estimate). Detecting birds on the outskirts of the tracking area is therefore still possible


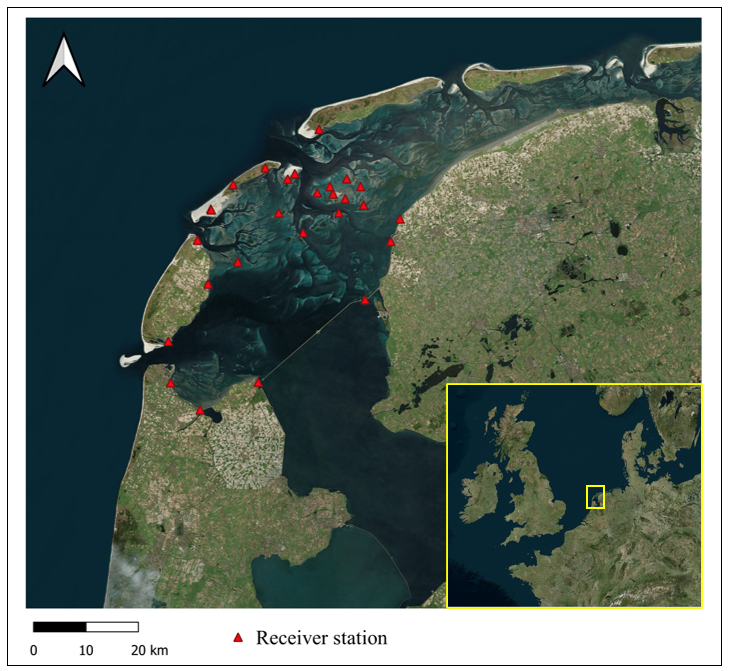


**Supplementary Figure S1.** Study area in the Dutch Wadden Sea with 26 receiver stations of the WATLAS-system (red triangles).

**Data S2**

**
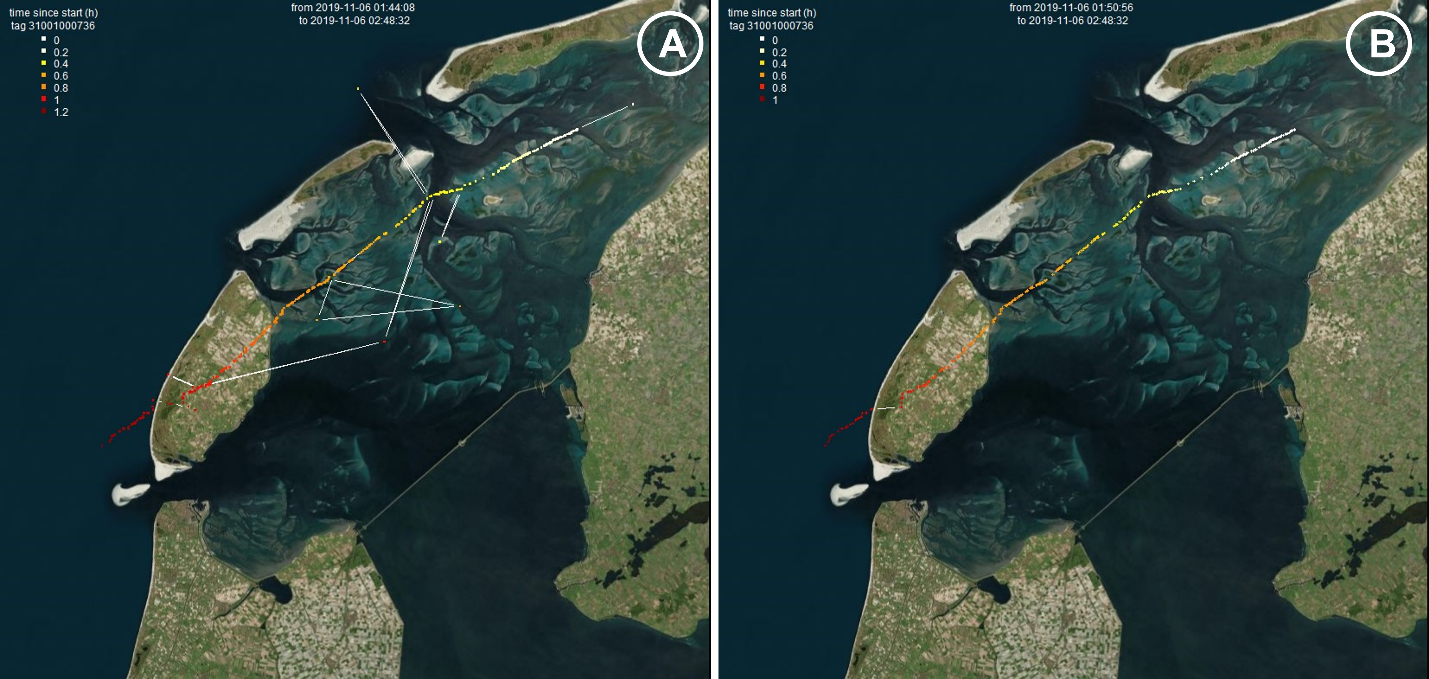
**

**
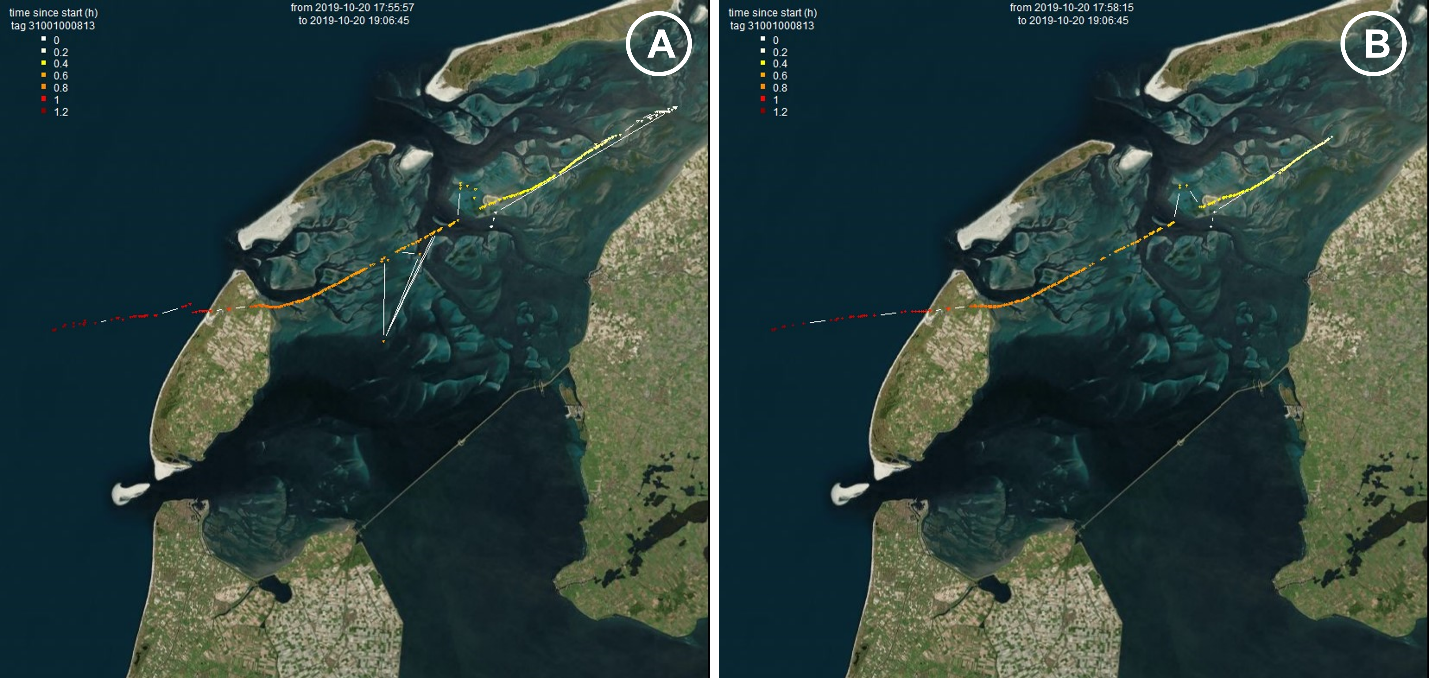
**

**Supplementary Figure S2.1** (A) Unfiltered and (B) filtered tracks of two individuals that left on westward relocation flights.

**Data S3**

We used to following formula to calculate wind assistance:

$Wind assistance (WA)=y cos\theta$ (1)

Here, $y$ is equal to the wind speed (ms^-1^), and $\theta$ represents the difference in degrees between the wind direction and a bird’s heading (Shamoun-Baranes *et al.* 2007, Kemp *et al.* 2012). We first calculated a bird’s air speed (the speed a bird flies using its own power), based on the bird’s ground speed (the speed we measure, which is affected by wind speed and direction), flight track direction, wind direction and wind speed. We measured a bird’s ground speed as the average speed of the first 20 minutes after its departure time. Then we used the flight direction and airspeed to calculate its heading. The heading is slightly different from the flight track direction we create from the coordinates, as the heading is the direction a bird would fly if there was no wind affecting its flight direction. All departing birds had headings ranging from southwest to northwest.

**Data S4**

**
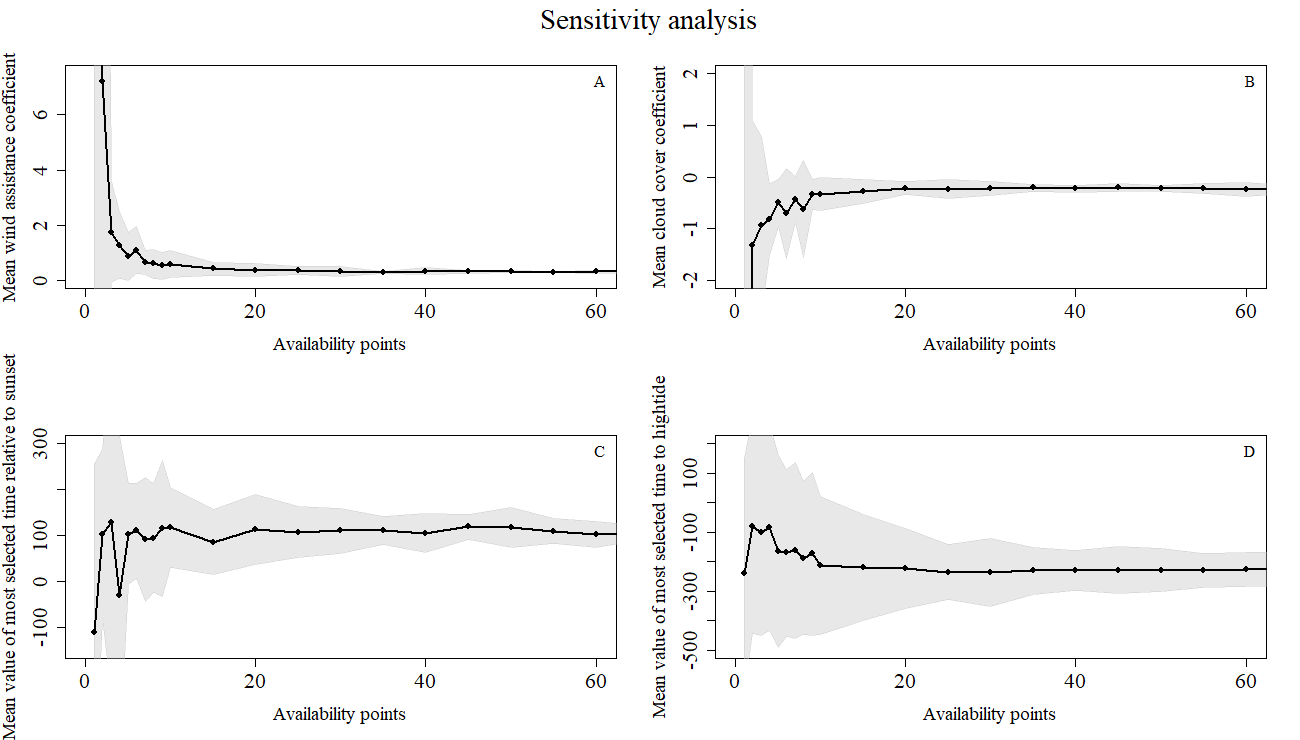
**

**Supplementary Figure S4.** We performed several sensitivity analyses to determine the number of randomly selected availability time-points in our RSF, by bootstrapping our model 100 times for each number of availability. We used the β-coefficient of tailwind assistance and cloud cover as parameter estimates,). For non-linear parameters, we extracted the estimate of time relative to high tide and time relative to sunset, which was most selected by our model (the peak value). The black dots represent the mean parameter estimate, where the grey polygon represents 95% confidence interval. We see mean values levelling off after approximately 15 availability points. For a more conservative approach, we chose 30 availability points (Northrup *et al.* 2013).

**Data S5**

**
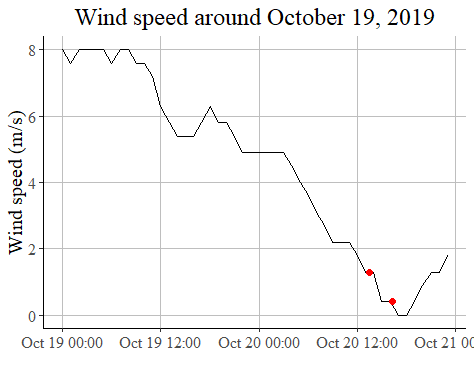
**

**Supplementary Figure S5**. Wind speeds from October 19, 2019 until October 21, 2019. The red dots indicate two departing birds that departed before sunset. The day before their departure time, wind speeds were high and unfavorable for the red knots. This result suggest that they have waited for the best first available wind conditions, even though this was before the sun had set.

**References**

**Beardsworth, C.E., Gobbens, E., van Maarseveen, F., Denissen, B., Dekinga, A., Nathan, R., Toledo, S. & Bijleveld, A.I.** 2022. Validating ATLAS: A regional-scale high-throughput tracking system. *Methods Ecol Evol* **13**: 1990–2004.

**Bijleveld, A.I., van Maarseveen, F., Denissen, B., Dekinga, A., Penning, E., Ersoy, S., Gupte, P.R., de Monte, L., ten Horn, J., Bom, R.A., Toledo, S., Nathan, R. & Beardsworth, C.E.** 2022. WATLAS: high-throughput and real-time tracking of many small birds in the Dutch Wadden Sea. *Animal Biotelemetry* **10**.

**Kemp, M.U., Shamoun-Baranes, J., van Loon, E.E., McLaren, J.D., Dokter, A.M. & Bouten, W.** 2012. Quantifying flow-assistance and implications for movement research. *J Theor Biol* **308**: 56–67.

**Northrup, J.M., Hooten, M.B., Anderson, C.R. & Wittemyer, G.** 2013. Practical guidance on characterizing availability in resource selection functions under a use-availability design. *Ecology* **94**: 1456–1463.

**Shamoun-Baranes, J., Van Loon, E., Liechti, F. & Bouten, W.** 2007. Analyzing the effect of wind on flight: Pitfalls and solutions. *Journal of Experimental Biology* **210**: 82–90.
